# Microtubule-Targeting Agents: Advances in Tubulin Binding and Small Molecule Therapy for Gliomas and Neurodegenerative Diseases

**DOI:** 10.3390/ijms26157652

**Published:** 2025-08-07

**Authors:** Maya Ezzo, Sandrine Etienne-Manneville

**Affiliations:** Cell Polarity, Migration and Cancer Unit, Institut Pasteur, UMR3691 CNRS, Université de Paris, Equipe Labellisée Ligue Contre le Cancer 2023, F-75015 Paris, France; maya.ezzo@pasteur.fr

**Keywords:** cytoskeleton, tubulin, cancer, neurodegeneration

## Abstract

Microtubules play a key role in cell division and cell migration. Thus, microtubule-targeting agents (MTAs) are pivotal in cancer therapy due to their ability to disrupt cell division microtubule dynamics. Traditionally divided into stabilizers and destabilizers, MTAs are increasingly being repurposed for central nervous system (CNS) applications, including brain malignancies such as gliomas and neurodegenerative diseases like Alzheimer’s and Parkinson’s. Microtubule-stabilizing agents, such as taxanes and epothilones, promote microtubule assembly and have shown efficacy in both tumour suppression and neuronal repair, though their CNS use is hindered by blood–brain barrier (BBB) permeability and neurotoxicity. Destabilizing agents, including colchicine-site and vinca domain binders, offer potent anticancer effects but pose greater risks for neuronal toxicity. This review highlights the mapping of nine distinct tubulin binding pockets—including classical (taxane, vinca, colchicine) and emerging (tumabulin, pironetin) sites—that offer new pharmacological entry points. We summarize the recent advances in structural biology and drug design, enabling MTAs to move beyond anti-mitotic roles, unlocking applications in both cancer and neurodegeneration for next-generation MTAs with enhanced specificity and BBB penetration. We further discuss the therapeutic potential of combination strategies, including MTAs with radiation, histone deacetylase (HDAC) inhibitors, or antibody–drug conjugates, that show synergistic effects in glioblastoma models. Furthermore, innovative delivery systems like nanoparticles and liposomes are enhancing CNS drug delivery. Overall, MTAs continue to evolve as multifunctional tools with expanding applications across oncology and neurology, with future therapies focusing on optimizing efficacy, reducing toxicity, and overcoming therapeutic resistance in brain-related diseases.

## 1. Introduction

Brain pathologies encompass a wide array of conditions that impair the structure and function of the central nervous system (CNS), ranging from highly proliferative tumours to progressive neurodegenerative diseases. Despite their diverse aetiologies and clinical expressions, many of these disorders converge on the disruption of cellular architecture and intracellular transport, processes critically governed by the cytoskeleton, particularly microtubules.

**Figure 1:**
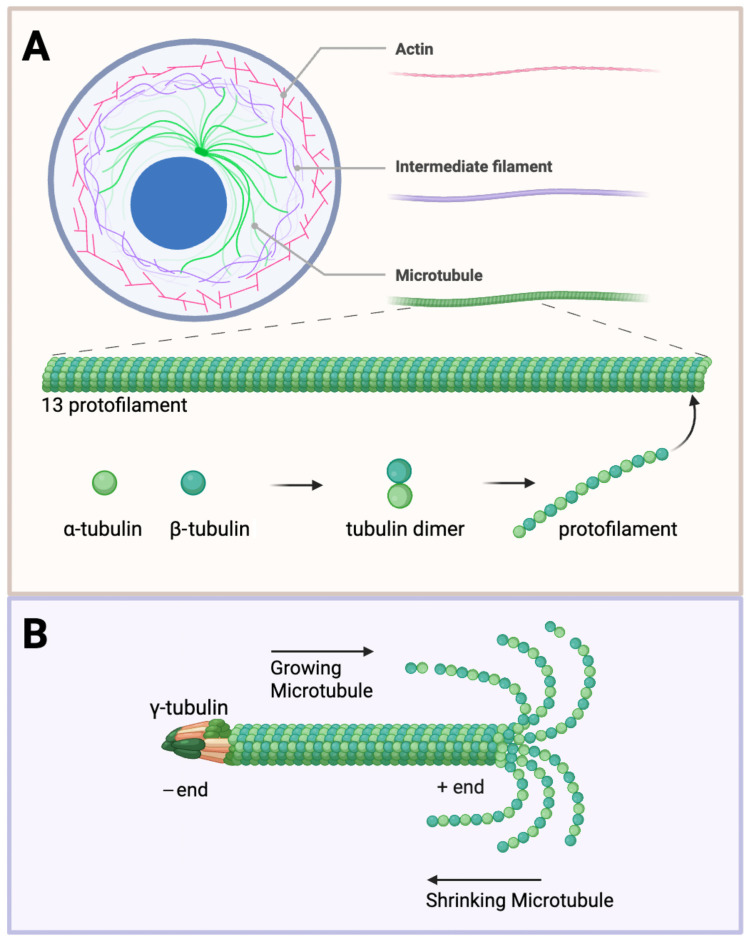
Microtubule structure and dynamics. (**A**) Cytoskeleton components and microtubule structure. The cell cytoskeleton is composed of actin (red), intermediate filaments (purple), and microtubules (green). Tubulin heterodimers consisting of α- (light green) and β-subunits (dark green) assemble into protofilaments. Thirteen protofilaments further polymerize to form a hollow cylinder about 25 nm in diameter. (**B**) Structural dynamics of microtubules. The γ-tubulin ring complex is the starting point for microtubule nucleation. Growing microtubules favour assembly at the plus end (left to right) versus shrinking towards the minus end (right to left). The plus end exposes the β-tubulin, and the minus end exposes the α-tubulin. Created with BioRender.com (accessed 25 June 2025).

A growing body of evidence links cytoskeletal remodelling as a central factor in both tumour progression and neuronal dysfunction. The cytoskeleton is a complex structural network comprising actin filaments, intermediate filaments, and microtubules (Figure 1). All are essential for maintaining the cellular architecture and facilitating dynamic processes such as mitosis, migration, and signal transduction. Among these, microtubules are particularly vital due to their highly dynamic nature and their central role in both proliferative and differentiated cell functions. In neurodegenerative diseases, such as Alzheimer’s and Parkinson’s, microtubule dysfunction leads to impaired axonal transport and synaptic degeneration, often exacerbated by abnormal tau hyperphosphorylation and aggregation, which destabilizes microtubule networks [1,2,3]. Conversely, in glioblastomas and other brain tumours, microtubule dynamics are hijacked to promote uncontrolled proliferation, invasion, and resistance to therapy [4,5]. The dual role of microtubules in both degenerative and neoplastic contexts emphasizes their significance in brain health and disease. Therefore, targeting microtubule behaviour—whether by stabilizing or disrupting their dynamics—holds promise for diverse therapeutic applications across the spectrum of brain pathologies. These impairments are often driven by the pathological modification or misfolding of microtubule-associated proteins (MAPs), such as tau, which aggregate and disrupt the microtubule network. Despite the differing clinical outcomes of these pathologies, both are united by a common thread: the pathological remodelling of the cytoskeleton, particularly microtubules.

Microtubules are ever-changing and fundamental to the structure and function of healthy brain cells; microtubules are abundant and can represent up to 10% of the total protein content in the brain [6]. They support neuronal morphology, aid intracellular transport, and enable synaptic function and plasticity for cell communication [7,8]. Microtubule regulation plays a central role in establishing diverse cellular architectures, including astral arrays, polar fibres, and mitotic spindles, that are critical for proper neuronal development and brain function. In neural progenitors, astral microtubules, anchored at centrosomes and extending toward the cortex, are essential for spindle orientation, influencing symmetric versus asymmetric divisions that shape cortical organization [9]. In oligodendrocytes, microtubules are crucial for extending processes and building myelin sheaths, often nucleating outside the centrosome at Golgi outposts. In astrocytes and microglia, dynamic microtubule networks support cell branching, motility, and responses to environmental cues, enabling glial cells to adapt and interact with neurons and their surroundings. In fact, mature, differentiated neurons are post-mitotic, meaning they no longer divide and rely on the support from glial and vascular cells for structural integrity and function [10,11]. Microtubules in brain vascular cells play a vital role in maintaining the integrity and function of the blood–brain barrier; they support endothelial junctions, regulate actin-mediated cytoskeletal tension, and enable proper trafficking [12,13]. An understanding of the roles and regulations of microtubules can provide insights into pathological conditions affecting the brain and beyond. Microtubule-targeting agents (MTAs) such as taxanes and vinca alkaloids have been employed in other cancers, but their use in brain cancers remains limited by issues like blood–brain barrier (BBB) penetration and neurotoxicity.

This review centralizes the role of microtubules in brain cancer and neurodegeneration, integrating recent insights into tubulin structural diversity (a new 9th tubulin binding site), brain-specific microtubule functions, and the translational challenges of CNS drug delivery, to inform the development, optimization, and translational deployment of MTAs. By exploring these intersections, we aim to provide a comprehensive overview that highlights the therapeutic relevance of cytoskeletal modulation in human health and disease.

### 1.1. Key Microtubule Functions: Cell Division and Migration

The process of cell division (mitosis) for growth and development, repair, reproduction, and preservation of genetic identity is essential. In fact, most of our knowledge about microtubule organization stems from studying dividing cells in 2D cultures. During mitosis, microtubules, normally found in a cytoplasmic display, reorganize to form a bipolar mitotic spindle, responsible for segregating chromosomes into two daughter cells. The formation of the mitotic spindle is highly regulated through the organization of microtubules and MAPs. It is composed of antiparallel microtubules anchored at the spindle poles with plus ends extending toward the chromosomes, relying on the dynamic instability of microtubule ends, driven by guanosine triphosphate (GTP) hydrolysis, to enable rapid remodelling during cell division. Additionally, the polarized microtubule lattice serves as a track for motor proteins like dynein and kinesins, which harness energy to generate directional forces essential for spindle function and chromosome movement [14,15].

The microtubules modulate key processes of migration through cell protrusions, adhesions, contractions, and intercellular transport [16,17,18]. They serve as tracks for intracellular transport, delivering essential components like membrane vesicles and signalling molecules to the leading edge of migrating cells. This targeted delivery is crucial for the formation of protrusions and the establishment of cell polarity. Microtubule interaction with focal adhesions facilitates their turnover, thus enabling the dynamic attachment and detachment necessary for cell movement. Microtubules not only facilitate the structural reorganization required for cell movement but also help coordinate the trafficking of vesicles and signalling molecules critical for adhesion turnover and guidance cues [19]—functions that are especially vital in the dense and polarized architecture of the brain. Their contribution to the spatial organization of signalling pathways regulates the cytoskeleton, particularly in astrocytes [20].

Microtubule involvement in cell migration varies significantly across cell types, depending largely on their adhesion dynamics. Highly adherent glial cells, such as astrocytes and microglia, depend on microtubules to regulate cell polarization, maintain directional persistence, and coordinate rear-end retraction during migration [21,22,23,24]. The multifaceted roles of microtubules across various brain cell types underscore their centrality in both the healthy functioning and pathological transformation of the central nervous system. From orchestrating mitotic spindle formation and intracellular transport to maintaining the neuronal architecture and enabling glial plasticity, microtubules act as dynamic scaffolds that integrate mechanical support with signalling control. Their dysfunction is not merely a consequence but often a driver of disease, contributing to tumour progression, therapeutic resistance, and neurodegeneration through altered dynamics, structural instability, or misregulated interactions with associated proteins and organelles.

### 1.2. Microtubule Malfunction in Brain Cancer and Neurodegeneration

In the CNS, the precise organization and regulation of microtubules are vital for neuronal and glial cell functions. Disruptions in microtubule dynamics and organization have been implicated in the pathogenesis of various brain cancers and neurodegenerative diseases.

Primary brain tumours develop from cells within the CNS. Gliomas represent about 25% of brain tumours in adults and account for most deaths from primary brain tumours [25]. Gliomas develop from glial or precursor cells and include glioblastoma, astrocytoma, oligodendroglioma, ependymoma, and oligoastrocytoma (mixed glioma) [25]. They are characterized by their high invasion and spreading [26,27], heterogeneity [28], and resistance against surgical resection, radiotherapy, and chemotherapy [29,30].

In brain tumours, particularly gliomas, microtubule nucleation and organization alterations contribute to tumour progression and invasiveness. The γ-tubulin ring complex (γ-TuRC) has been found to be dysregulated in cancer cells, leading to abnormal microtubule formation and chromosomal instability [reviewed in [31]]. Such dysregulation promotes uncontrolled cell proliferation. Mutations in tubulin genes, such as TUBA1A and TUBB3, have been associated with impaired microtubule dynamics, affecting cell division and contributing to tumorigenesis [32,33]. In pathological conditions, such as glioblastoma, the regulation of microtubule dynamics (with an emphasis on MAPs and post-translational modifications (PTMs)) becomes abnormal, leading to enhanced migratory capabilities of cancer cells [34,35].

Dysregulation of centrosomal or cortical microtubule regulators has been directly linked to microcephaly and mitotic failure in the developing brain [36,37]. Notably, microtubule polarity and dynamics are often hijacked in brain tumours such as glioblastoma. The unusual stabilization or reorganization of microtubules can support uncontrolled proliferation, mitotic slippage, and chromosomal instability—hallmarks of cancer progression [38]. Thus, both the normal patterning and pathological disruption of microtubule polarity play pivotal roles in brain development and oncogenesis.

A key facilitator of therapeutic resistance is network formations between large cell populations of glioblastoma cells connected by tumour microtubes [29]; tumour microtubes contain components of the cytoskeleton, including microtubule αβ-tubulin dimers [39]. These tumour microtubes enable long-range calcium signalling and organelle trafficking between glioma cells, enhancing their survival and repair after damage. Molecular targets such as GAP-43, Connexin 43, and tweety-homolog1 (TTYH1) are critical for the formation and function of tumour microtubes [40,41].

In neurodegenerative diseases, microtubule dysfunction is a shared pathological feature [42]. For instance, in Alzheimer’s disease, hyperphosphorylation of the microtubule-associated protein tau leads to its detachment from microtubules, resulting in microtubule destabilization and impaired axonal transport [43], reduced levels of acetylated α-tubulin [2], and increased deacetylating enzyme histone deacetylase (HDAC) 6, in degenerating brain regions [44]. Similarly, in Parkinson’s disease, reduced acetylation of α-tubulin correlates with defective axonal transport and neuronal degeneration [45]. Moreover, mutations in tubulin genes encoding microtubule-interacting proteins, Parkin and leucine-rich repeat kinase 2 (LRRK2) [46], have been linked to various neurodevelopmental disorders, including Parkinson’s disease, highlighting the importance of proper microtubule regulation in CNS development and function.

Given their pervasive influence on cellular behaviour in the brain, microtubules present both a challenge and an opportunity in therapeutic design. MTAs, while potent in other malignancies, require nuanced adaptation for effective use in the CNS, particularly in overcoming barriers such as neurotoxicity and limited brain penetration. Continued exploration into the precise molecular determinants of microtubule regulation is thus crucial for advancing both our understanding and treatment of brain tumours and neurodegenerative diseases. To that end, the next section of this review will delve deeper into the structural and molecular landscape of microtubules, with particular emphasis on tubulin isoform diversity, post-translational modifications, and the specific binding sites exploited by MTAs—including the recently identified ninth binding site. This structural perspective provides essential context for rational drug design and highlights how the intricate architecture of microtubules informs their functional specificity and therapeutic modulation.

## 2. Microtubules: Structure and Tubulin Binding Sites

Microtubules are the largest and most rigid [47] of the filamentous cytoskeleton, with a diameter of 25 nm, as compared to actin filaments with a diameter of about 6 nm and intermediate filaments at about 10 nm [48]. These extensive filaments are formed by tubulin heterodimers that assemble into protofilaments, subsequently integrating 13 protofilaments into a hollow, cylindrical tube—a highly dynamic process. The head-to-tail organization of tubulin dimers forms a polarized polymer with two distinct ends—the minus ends and the plus ends [49] (Figure 1). The rapid polymerization and depolymerization at the free ends (ordinarily the plus ends) is termed dynamic instability [50], regulated by the binding and hydrolysis of GTP. The minus ends are generally anchored to structures known as microtubule-organizing centres (MTOCs), with the prime MTOC in cells being the centrosome, from which they tend to grow outward towards the plasma membrane. The growing and shrinking of microtubules exert forces that shift subcellular structures, especially in cell division. With this knowledge, many effective anti-proliferative drugs bind to tubulin and interfere with the dynamics of microtubules [51].

### 2.1. Microtubule Organization and Regulation

Tubulin, a 50 kDa globular protein that binds GTP, is the fundamental building block of microtubules. It exists in six isoforms: alpha (α), beta (β), gamma (γ), delta (δ), epsilon (ε), and zeta (ζ) [52,53]. In all eukaryotic cells, microtubules are assembled predominantly from αβ-tubulin heterodimers. These heterodimers typically organize into radial arrays anchored at the centrosome, forming an aster-like microtubule network characteristic of centrosome-driven organization [54]. However, a range of microtubule organizations exists across cell types in the brain. For instance, axons contain uniformly oriented microtubules with plus ends directed outward, whereas dendrites include a mixed population of polarity with many minus-end-out microtubules; neural progenitor cells display highly organized networks of microtubules for a more elongated shape; oligodendrocytes organize radial microtubules for local branching and lamellar microtubules to facilitate extensions to wrap around axons; the external environment heavily influences astrocytes, therefore microtubules must adapt their changes in cell shape and extensions; and microglia, resident immune cells in the brain, significantly alter microtubule organization depending on their resting or activated state [10].

The most common template for de novo microtubule formation is the γ-TuRC, the nucleator, acting as the foundation for tubulin heterodimers [55]. One may even ask how microtubules, which are nearly identical αβ-tubulin heterodimers, can produce various arrays of functions; one rather simple response—microtubules do not work unaided. In the complex environment of the brain, PTMs of tubulin play a pivotal role in shaping microtubule behaviour, orchestrating the cytoskeletal dynamics essential for development, synaptic function, and cellular plasticity. Some PTMs include acetylation [56], detyrosination [57], polyglutamylation [58], polyglycylation [59], and phosphorylation [60]. These modifications can directly influence the stability and structure of microtubules; for instance, tubulin acetylation can enhance microtubule flexibility, preventing structural damage [61]. PTMs can also indirectly influence microtubules by interacting with MAPs, such as tau, MAP2, and kinesins, which bind to microtubules and influence their dynamics, transport functions, and cellular positioning [62,63].

### 2.2. Structural Support and Intracellular Organization

Microtubules are critical for maintaining cell integrity, particularly in complex and mechanically demanding environments such as the brain [64]; they stand out for both their diameter and mechanical strength. Microtubules maintain the cell shape and can resist compression [65,66], enabling them to withstand various mechanical stresses. They can also interact with actin and myosin stress fibres, forming an integrated network that is crucial for cell migration and morphogenesis, processes particularly relevant in the developing brain [20,67].

In brain cells, microtubules are crucial for ensuring the overall architecture of neurons and glial cells [68,69,70]. Microtubules are particularly important for neurons, and their ability to resist bending and compressive forces is important in the brain, where neurons are exposed to mechanical stress during development and synaptic plasticity [7,66,71]. In addition, the ability of microtubules to endure the stresses associated with long-range transport is essential, particularly in neurons, where vesicles, organelles, and protein complexes must be shuttled along axons and dendrites [72].

Microtubule-based transport is essential for neuronal function, facilitating the long-distance movement of various cellular components, including proteins, lipids, mitochondria, and synaptic vesicles. Acting as roads for motor proteins like kinesins and dyneins, microtubules facilitate the directed movement of organelles, vesicles, and macromolecules within the cell [15,73,74]. Each motor typically moves on microtubules only in one direction—towards the plus (kinesin) or the minus end (dynein) [75,76]. The geometry and directionality of microtubules determine the patterns of intracellular transport and guide organelle positioning. In glial cells, cargo transport plays a crucial role in maintaining cellular function and health. Oligodendrocytes rely on the transport of endosomes and exosomes to deliver essential myelin proteins and engage in signalling with neurons [77,78]. Astrocytes depend on efficient cargo transport, including excitatory amino acid transporter 1 (EAAT1), and connexin 43, the latter playing a significant role in glioma invasiveness [79,80]. Microtubule-based transport also includes the movement of intermediate filaments [81], shown to be required for glioblastoma invasion [82].

### 2.3. Tubulin Binding Sites

**Figure 2:**
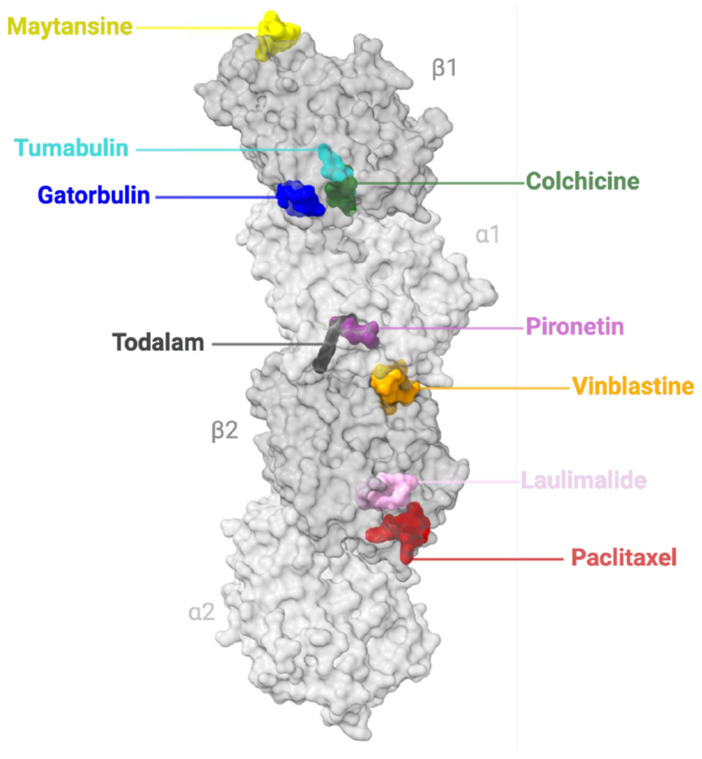
Binding sites of Microtubule-Targeting Agents (MTAs). Nine MTA binding sites of tubulin. Two tubulin heterodimers (α-subunits, light grey; β-subunits, grey). The representative agents bound to the nine sites are shown in colour: maytansine site (yellow, PDB ID 4TV8); tumabulin site (cyan, PDB ID 7CEK); gatorbulin site (blue, PDB ID 7ALR); colchicine site (green, PDB ID 4O2B); pironetin site (purple, PDB ID 5LA6); todalam site (black, PDB ID 7Z7D); vinca site (vinblastine, orange, PDB ID 5J2T); peloruside A/laulimalide site (pink, PDB ID 4O4J); and taxane site (paclitaxel, red, PDB ID 6WVR). Schematic made with ChimeraX (version 1.10rc202506232245).

The electron crystallography of tubulin [83,84] has clarified how GTP binding and hydrolysis at β-tubulin regulate microtubule dynamics. GTP-bound tubulin promotes polymerization, while GDP-bound tubulin favours depolymerization, explaining microtubule instability [49,85]. Structural data also revealed binding sites for MAPs and drugs, improving our understanding of their effects on microtubule stability (Figure 2). Tubulin binding sites serve as key interaction hubs for numerous MTAs, allowing drugs to modulate microtubule dynamics by either stabilizing or destabilizing polymer formation. These sites are exploited by a wide range of therapeutic compounds [84,86,87].

There are 8 established binding sites of tubulin: colchicine [88], gatorbulin [89], laulimalide [90], maytansine [91], taxane [92], pironetin [93], todalam [94], and vinca site [95]. However, very recently, a 9th binding site was identified as tumabulin in a study by Li et al. [96], and 10 additional binding sites have been predicted through a combination of computational modelling and crystallographic fragment screening [86]. Mühlethaler et al. revealed an intricate network of binding pockets for small molecules to target, where 56 chemically diverse portions could potentially bind to 10 distinct sites in these pockets [86]. These sites could possibly pass beyond altering tubulin polymerization and interfere with MAP interactions as well.

The colchicine site is in the middle domain of β-tubulin as a deep pocket [84], further subdivided into a main zone and two additional accessory pockets either facing α-tubulin or deeper in the β-tubulin subunit [88,97]. The gatorbulin site lies between αβ-tubulins at their intradimer interface [89]. The site of laulimalide is near the adjacent interfaces of protofilaments on the outer microtubule surface and may inhibit microtubule disassembly by clamping protofilaments together [98,99,100]. The maytansine domain can be found on the exposed β-tubulin pocket, found to bind independently of the conformational state of tubulin, resulting in the inhibition of longitudinal tubulin formation [91]. The taxane site is located in a pocket of β-tubulin, on the luminal side of microtubules, and can establish both hydrophobic and polar contacts; the taxane site ligands result in stabilized microtubules and a suppression of their dynamics [49,101]. The binding site of pironetin is exclusive to α-tubulin, and ligand binding reveals the binding pocket due to conformational change through an induced fit mechanism [93]. The todalam site is located between two lengthwise tubulin dimers, allowing it to bind to both α- and β-tubulin monomers of both tubulin dimers [94]. Comparably, the vinca site is at the inter-dimer interface between two aligned tubulin dimers, with a core zone and a pocket in reach of an exchangeable guanosine nucleotide site on β-tubulin [102]. The newfound tumabulin site exists at the interface of α1-tubulin, β1-tubulin, and stathmin-like protein B3 (RB3) [96] (Figure 2).

The structural and regulatory complexity of microtubules reveals how central they are to the cellular architecture, trafficking, and signalling within the brain. Their unique dynamic instability, coupled with intricate regulation by tubulin isoforms, PTMs, and MAPs, enables diverse cell type-specific architectures and functions. In both neurons and glial cells, microtubules not only maintain physical integrity but also serve as essential highways for intracellular communication. Critically, the discovery of multiple tubulin binding sites has paved the way for the development of a wide array of microtubule-targeting agents (MTAs), making the cytoskeleton not just a structural element but also a promising therapeutic target.

With a comprehensive understanding of microtubule architecture and regulation, we can now examine how this knowledge has been translated into therapeutic applications. The identification of specific tubulin binding sites has catalyzed the development of drugs that either stabilize or destabilize microtubules, disrupting cellular dynamics in ways that are especially valuable in the treatment of proliferative diseases such as cancer. The next section delves into these microtubule-targeting agents (MTAs), exploring their mechanisms of action, therapeutic utility, and relevance in modulating glial cell behaviour and brain pathology.

## 3. Microtubule-Targeting Agents—Stabilizers

Microtubules’ key role in cell division has made them a key target for anticancer therapies for decades. MTAs are among the most effective chemotherapeutics. They can act as stabilizers or destabilizers of microtubules to inhibit mitosis and induce cell death. Despite their effectiveness, these traditional MTAs often result in dose-limiting toxicities and the development of resistance, requiring the development of next-generation compounds.

Recent advances in structural biology and computational modelling (reviewed in [103]) have expanded our understanding of tubulin’s diverse binding pockets, revealing novel sites for therapeutic intervention beyond the classical taxane and vinca domains (Figure 2). This has accelerated the discovery of innovative MTAs with improved selectivity, unique mechanisms of action, and potential to overcome resistance. These newer agents aim to not only disrupt mitotic progression but also to modulate microtubule dynamics in non-dividing cells, opening the door to broader therapeutic applications, including neurodegenerative diseases. In this section, we explore the spectrum of MTAs as stabilizers—from well-established representatives to emerging small molecules (Table 1).

### 3.1. Microtubule-Stabilizing Agents

**Figure 3:**
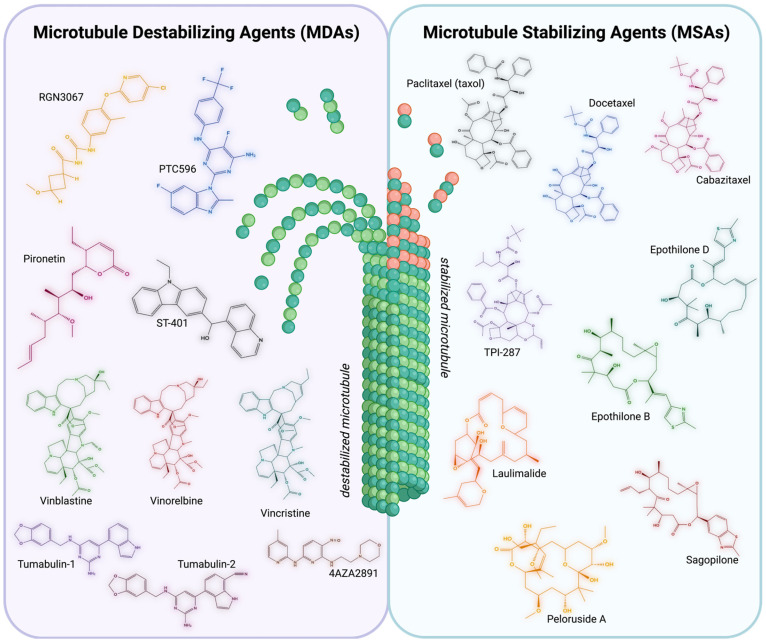
Summary of Microtubule-Targeting Agents. Microtubule-Destabilizing Agents (MDAs) [left]. Chemical structures of representative MDAs. MDAs aim to destabilize microtubules, leading to impaired structural integrity and inhibiting the tubulin polymerization into microtubules. RGN3067 (yellow), PTC596 (blue), Pironetin (pink), ST-401 (black), Vinblastine (green), Vinorelbine (red), Vincristine (teal), Tumabulin-1 (purple), Tumabulin-2 (dark pink), and 4AZA2891 (brown). Microtubule-Stabilizing Agents (MSAs) [right]. Chemical structures of representative MSAs. MSAs aim to stabilize microtubules, promoting the polymerization of purified tubulin and enhancing microtubule density. Taxol (black), Docetaxel (blue), Cabazitaxel (pink), TPI-287 (purple), Epothilone B (green), Epothilone D (teal), Laulimalide (orange), Peloruside A (yellow), and Sagopilone (red). Structures built with MolView (v4.10.0). Created with BioRender.com (accessed 25 June 2025).

Microtubule-stabilizing agents (MSAs), as the name suggests, tend to stabilize microtubules by promoting microtubule assembly (Figure 3). They show potential not only as anticancer therapies but also in addressing neurological disorders due to their ability to reinforce microtubule integrity.

As previously mentioned, the disruption of microtubule dynamics is a hallmark of several neurodegenerative diseases. These MSAs may help counteract disruptions by promoting microtubule polymerization and stability, thus supporting axonal transport and neuronal survival. Tau proteins normally stabilize axonal microtubules and support neural transport and growth. However, in tauopathies (i.e., Alzheimer’s and Parkinson’s), microtubule-associated tau becomes hyperphosphorylated and aggregates into insoluble filaments, losing its stabilizing ability [124,125]. This destabilization contributes to axonal dysfunction and cognitive deterioration. Since tau and some MSAs share the ability to bind to β-tubulin and stabilize microtubules [126,127], it is hypothesized that MSAs could compensate for the lost tau function. Their potential to treat neurodegenerative disease is largely targeted (reviewed in [128,129]).

In brain tumours, MSAs have emerged as a powerful tool by reducing microtubule dynamics, which are essential for proper mitotic spindle formation and successful cell division of cancer cells. In highly proliferative tumours, such as glioblastomas, this disruption of microtubule function can trigger prolonged mitotic arrest and apoptosis [87,104]. MSAs also offer several potential benefits beyond mitotic inhibition. The stabilization of microtubules can impair the cytoskeletal remodelling necessary for tumour cell migration and invasion [17,105], key features of aggressive brain tumours like glioblastoma and diffuse midline gliomas [130]. Additionally, microtubule stabilization can influence intracellular trafficking and signalling pathways that support tumour growth and resistance to therapy [5,111].

However, applying agents in the CNS, for both cancers and neurological diseases, poses significant challenges, such as limited BBB permeability and potential neurotoxicity. Ongoing research continues to explore novel MSAs with better BBB permeability and selectivity for more effective and targeted therapies.

#### 3.1.1. Taxane-Site

Taxanes are the most well-known MSAs and have demonstrated efficacy in many malignancies. The first MSA to be discovered was paclitaxel (Taxol^®^) [131], initially thought of as a mitotic phase targeting drug, but later found to target microtubules throughout the cell cycle [132]. Taxol^®^ belongs to the taxane family and shares the binding site with similar taxane docetaxel (semi-synthetic analogue of paclitaxel [133]) and even non-taxane epothilone [134] (Figure 2). The main mechanism of action is through binding to tubulin to inhibit the disassembly of microtubules, promoting cell death [106]. The treatment with Taxol^®^ in tau transgenic mice improved motor function and restored fast axonal transport by increasing microtubule stability, supporting the hypothesis of compensating for tau loss [135].

The treatment with Taxol^®^ of malignant gliomas and brain metastases has been reported [136,137]. Other taxane equivalents, including ortataxel [138], cabazitaxel [139], and TPI-287 [107], have also been assessed. TPI-287 showed a well-tolerated response in three out of seven patients of reoccurring glioblastoma reported in 2014, with an updated report of the phase I trial of safety and efficacy for TPI-287 ten years later [108]; however, a randomized trial of TPI-287 in Alzheimer’s patients was less tolerated due to anaphylactoid reactions [109].

However, to target brain tumours, Taxol^®^ and its derivatives require aid to cross the BBB [110]. To solve this issue, the intranasal injection of drugs is the fastest route to circumvent the BBB, but with the addition of a vehicle to transport paclitaxel, greatly improves the delivery [112]. Promising methods include nanomedicines, nanoparticles, nanoemulsions, and nanostructured lipids [113,140]; Abdel-Haq et al. demonstrated that paclitaxel delivery with nanoparticles, both intranasally and intravenously, resulted in considerable paclitaxel in the brain of rats and no toxicity [114].

Among the known MSAs, epothilones have shown promise for neurological diseases and brain tumours due to their ability to cross the BBB—a limitation of paclitaxel and its equivalents [114,115]. Many derivatives of epothilones have been synthesized to target many cancers [116]. Patupilone (epothilone B) was found to inhibit the migration of cells of glioblastomas without affecting the growth and shortening rates of microtubules [117]. Sagopilone (ZK 219477) has been tried and well tolerated in a phase II clinical trial for recurrent glioblastoma, where this synthetic analogue of epothilone B induces apoptosis in cells by inhibiting microtubule depolarization [118], but no evidence of relevant antitumour activity was found.

Epothilone D, effective at low doses, enhances axonal microtubule density, reduces axonal dystrophy, and improves cognitive performance in mouse models of Alzheimer’s disease [119,120]. In MPTP-induced models of Parkinson’s, epothilone D reversed microtubule damage and supported axonal repair [121]. Likewise, epothilones have shown benefits in other CNS maladies through models of stroke and spinal cord injury, where they promote axonal regrowth and improve functional recovery [122,141].

#### 3.1.2. Laulimalide-Site

As an alternative to taxanes, nontaxane MSAs bind at the laulimalide site of tubulins on the external surface [123] (Figure 2). The anticancer properties studied have shown that the synthetic laulimalide exhibited powerful in vitro cytotoxicity against various cancer cell lines [142]. As no studies have yet explored laulimalide’s efficacy against brain tumours, it leaves potential in the oncology field. However, nontaxane MSAs, including laulimalide and peloruside A, have been demonstrated to function regardless of tau overexpression, shown in a mouse N2a model of neuroblastoma cells [143], and peloruside A could even restore axonal outgrowth and branching in rat cerebral cortex neurons [144].

In summary, MSAs represent a promising class of compounds with therapeutic potential that extends beyond traditional anti-mitotic applications, offering new avenues for treating both aggressive brain tumours and neurodegenerative diseases. While challenges such as limited brain penetration and toxicity remain, ongoing innovation—particularly with non-taxane MSAs and advanced delivery systems—continues to push the boundaries of what is possible.

We now turn to the complementary class of microtubule-targeting agents: microtubule-destabilizing agents (MDAs). These compounds, which inhibit microtubule polymerization, have shown distinct advantages in targeting proliferative tumour cells and overcoming resistance mechanisms, warranting further exploration.

## 4. Microtubule-Targeting Agents—Destabilizers

MDAs have acquired renewed interest, not only for their ability to disrupt mitosis in rapidly dividing cells, such as glioblastoma, but also for their emerging relevance in modulating microtubule dynamics in post-mitotic neurons. This expanded therapeutic scope positions destabilizing MTAs as promising candidates across both oncological and neurodegenerative contexts. In the following section, we focus on the landscape of microtubule-destabilizing compounds, from well-characterized agents to novel small molecules currently under investigation (Table 2).

### 4.1. Microtubule-Destabilizing Agents

In brain tumours, cancer cells exploit hyperactive microtubule dynamics to support uncontrolled growth, invasive behaviour, and resistance to therapy. MDAs disrupt these dynamics by binding to tubulin and impairing polymerization (Figure 3), leading to mitotic arrest and cell death [156]. However, in the context of neurological diseases, especially those involving tau dysfunction, MDAs are less favoured due to their potential to further disrupt already compromised microtubule networks. Thus, for most neurodegenerative and CNS repair strategies, microtubule stabilization remains the preferred therapeutic approach. Several MDAs have shown efficacy in preclinical and clinical studies targeting malignant CNS tumours. However, their application, much like MSAs, is limited by poor BBB penetration and neurotoxicity.

#### 4.1.1. Colchicine-Site

Ligand interactions with colchicine involve mainly hydrophobic contacts as well as a few polar contacts and result in microtubule formation inhibition by preventing a straight conformational change in tubulin [88,145] (Figure 2). These compounds, especially those that have a 3′,4′,5′-trimethoxyphenyl substitution, demonstrate effective microtubule disruption, and some derivatives have been shown to exhibit IC_50_ values as low as 0.6 nM in cell lines [146].

The 3-nitropyridine analogues have emerged as a novel group of colchicine-site binders; their actions target G2-M phase cell cycle arrest and inhibit tubulin polymerization [147]. The synthesis of 4AZA2891, although not tested specifically for brain cancer, was found to reach the brain in mice [147]. Despite the efficacy in both in vitro and in vivo models, neurotoxicity remains a concern for clinical development.

ST-401 is amongst those that stand out due to its brain-penetrant properties and its unique mechanism of action. ST-401 inhibits tubulin assembly by binding to the colchicine site, killing cancer cells in the interphase rather than during mitosis [148]. Its ability to penetrate the BBB and its effectiveness against glioblastoma cells further emphasize the potential of colchicine-site MDAs.

Similarly, PTC596, an investigational small-molecule tubulin-binding agent, has shown promising preclinical and early clinical development, particularly in glioblastoma [149]. It is distinct from other tubulin-binding agents due to its high oral bioavailability and ability to effectively cross the BBB. Through crystallographic studies, it was revealed that PTC596 binds to the colchicine site on tubulin while lacking the trimethoxyphenyl moiety, distinguishing its binding profile from other agents. This binding also leads to microtubule destabilization, G2/M cell cycle arrest, and apoptosis in cancer cells without the neuropathy commonly associated. In preclinical models, PTC596 demonstrated inhibited growth of various tumour types, including glioblastoma. Notably, in an orthotopic glioblastoma mouse model, PTC596 monotherapy significantly prolonged survival, even when treatment was delayed, whereas temozolomide, a standard therapy, showed no efficacy. Ongoing phase I clinical trials are evaluating PTC596 in combination with radiation therapy for pediatric diffuse intrinsic pontine glioma (DIPG) [150].

A more recent small molecule, RGN3067, designed to also penetrate the BBB, exhibits low molecular weight, low polar surface area, and a high CNS multiparameter optimization (MPO) score [151]. Its efficacy both in vitro and in vivo was tested, showing induced G2/M cell cycle arrest in glioblastoma cell lines, and suppressed growth of patient-derived glioblastoma xenografts in mice.

#### 4.1.2. Pironetin-Site

Pironetin-site MDAs represent the exclusive binding to the pocket on α-tubulin (Figure 2); pironetin and its equivalents modify a specific cysteine residue (Cys316) on α-tubulin [152]. This interaction disrupts the polymerization of tubulin, leading to impaired mitotic spindle formation and thus cell cycle arrest. Vogt et al. found that the effects of pironetin displayed mitotic arrest and programmed cell death on a glioblastoma cancer cell line (T98G) [153]. Although research on pironetin is still in the early stages, these studies show promise as anticancer agents, with potential advantages such as reduced resistance compared to existing therapies [153,154].

#### 4.1.3. Vinca Domain

Vinca alkaloids target the vinca-binding site on β-tubulin, which interferes with the polymerization of microtubules, thereby inhibiting the formation of the mitotic spindle and halting cell division [155] (Figure 2). Vincristine has been one of the most widely studied vinca alkaloids in the treatment of gliomas, particularly in combination therapies (discussed below). It is frequently used in brain tumour treatment regimens, such as those for medulloblastomas and glioblastomas [157,158].

Vinblastine and vinorelbine are also used in the treatment of gliomas, although they are less commonly used than vincristine in clinical settings. These drugs work similarly by disrupting microtubule dynamics and arresting cells in the metaphase, leading to apoptosis [159]. Vinorelbine has shown efficacy in treating glial tumours, including pediatric DIPG, where it was successful, in combination with nimotuzumab, in lowering toxicity and improving quality of life [160].

#### 4.1.4. Tumabulin-Site

While quite fresh, the use of tumabulin shows some promise and hope for anticancer therapies. In their 2025 study, Li et al. identified the “Tumabulin site,” which mediates the interaction between tubulin and the stathmin-like protein RB3 [96] (Figure 2). This site is located at the interface of α1-tubulin, β1-tubulin, and RB3 within the tubulin–RB3–tubulin tyrosine ligase complex. The small molecule Tumabulin-1 (TM1), a derivative of BML284 (colchicine-site binding agent [161]), binds simultaneously to the familiar colchicine site and the newly revealed tumabulin site. Notably, two TM1 molecules bind cooperatively to this relatively large pocket, interacting with all three proteins. Essentially, this binding depends on the presence of RB3; it is absent when RB3 is missing. Furthermore, Li et al. designed and synthesized Tumabulin-2 (TM2), which selectively binds the tumabulin site without engaging the colchicine site. TM2 acts as a molecular glue, strengthening the interaction between RB3 and the tubulin dimer, thereby enhancing RB3′s tubulin-depolymerizing activity. These findings confirm the existence of a 9th tubulin-binding site and offer a promising foundation for developing tubulin to RB3 molecular bonds as a next generation of anticancer therapeutics.

MDAs continue to expand the therapeutic arsenal against brain tumours, with several novel compounds demonstrating promising brain penetration, reduced toxicity, and distinct mechanisms of action. Their ability to disrupt aberrant microtubule dynamics in proliferative tumour cells—especially glioblastomas—marks them as valuable tools in oncologic therapy. However, their neurotoxicity and limited efficacy as monotherapies—particularly in complex diseases like glioblastoma and DIPG—underscore the need for more comprehensive strategies. Increasingly, combination therapies are emerging as a compelling solution, designed to overcome therapeutic resistance and broaden the scope of action. The following section explores how MTAs are being integrated into multi-agent regimens—paired with radiation, immune modulators, or targeted drugs—to enhance treatment outcomes in both brain tumours and neurodegenerative disorders.

## 5. Combination Agents

Brain tumours and neurodegenerative diseases are highly complex and diverse, which makes treating them with a single therapy often insufficient for long-term benefit. Because of this, combination treatments—designed to target multiple disease pathways at once—are becoming more crucial for improving outcomes. In brain tumours like glioblastoma and DIPG, combining cytotoxic drugs with targeted therapies, immune modulators, or radiation helps to overcome resistance, kill more tumour cells, and reduce the risk of recurrence.

Combining MTAs with radiation, specific antigens, HDACs, or kinases may not only enhance cytotoxicity but also modulate the tumour microenvironment to reduce invasion and improve immune responsiveness. Combination therapy with vincristine, a vinca alkaloid, is recommended for gliomas [162]. A phase III study showed that patients with oligodendroglioma who received vincristine alongside radiation had longer progression-free survival, and long-term follow-up revealed significantly improved overall survival compared to those who received radiation alone [163]. The combination of TRX-E009-1, colchicine binding MDA, with HDAC inhibitor SAHA, enhanced by irradiation, resulted in reduced clonogenic survival of DIPG cells in vitro [164]. A phase I study of an oral colchicine MDA Lisavanbulin used in combination with standard radiation therapy was found to be safe up to a dose of 15 mg daily in patients with newly diagnosed, unmethylated glioblastoma [165].

In mouse xenograft studies using a patient-derived model and a U-87 MG human cell line model of glioblastoma, PTC596 in combination with paclitaxel was adequately tolerated and significantly more efficacious than the responses to the corresponding monotherapies [149].

Targeting both the colchicine binding site and other molecular targets can engage multiple pathways to achieve synergistic effects, enhancing tumour cell killing while minimizing adverse effects [166]. Puxeddu et al. synthesized pyrrole-based compounds, binding to the colchicine site and enzymes of the metabolism (in this case, human topoisomerases I and II), to inhibit tubulin polymerization and suppress the proliferation of glioblastoma cells [167]. Compound 7 has potential as a dual-targeting agent, demonstrated in BALB/c nu/nu mice injected with U87MG cells; compound 7 significantly inhibited tumour cell proliferation, tumorigenesis, and angiogenesis [167].

Research has increasingly focused on liposomal formulations that encapsulate the drugs to improve brain penetration and reduce systemic side effects [168]. Liposomal vincristine, for example, has been tested in various clinical trials and has shown increased efficacy in glioma treatment compared to the conventional drug [169]. Liposomal encapsulation not only enhances drug delivery to the tumour but also minimizes the neurotoxic effects associated with vincristine.

The combination of monoclonal antibodies with effective small-molecule drugs has created a class of targeted therapies known as antibody-drug conjugates (ADCs), more effective than previous heterogeneous ADCs [170]. Utilizing antibodies that specifically recognize tumour-associated antigens can minimize outside effects or damage to healthy tissues [171]. In recent years, several ADCs combining monoclonal antibodies with MTAs have been under investigation for glioma treatment.

AMG 595 is designed to target glioblastoma multiforme cells expressing the EGFRvIII mutant, a variant found in approximately 25–50% of glioblastoma multiforme cases. This ADC consists of a fully human anti-EGFRvIII monoclonal antibody conjugated to DM1, a potent maytansinoid MDA, via a non-cleavable linker. Upon binding to EGFRvIII on tumour cells, AMG 595 is internalized and trafficked to the lysosome, where DM1 is released, leading to microtubule destabilization and subsequent mitotic arrest, as evidenced by increased phospho-histone H3 levels in treated tumours [172]. In a phase I clinical trial involving patients with recurrent EGFRvIII-positive glioblastoma multiforme, AMG 595 was well-tolerated, with thrombocytopenia being the most common dose-limiting toxicity [172].

Patients with advanced tumours exhibiting EGFR overexpression or EGFRvIII mutation were tested with ADC depatuxizumab mafodotin (depatux-m), a monoclonal antibody targeting the EGFR linked with agent monomethyl auristatin F, which binds to the vinca domain of tubulin and disrupts microtubule dynamics. While generally well-tolerated, only a partial response was observed in one patient with EGFR amplified in 2018 [173], and no significant improvement in overall survival or new safety risks were identified for the depatux-m group in a 2022 study [174]. Overall, while depatux-m showed some activity in specific subgroups, it did not confer a survival advantage in the broader EGFR-amp glioblastoma population, suggesting that depatux-m may offer clinical benefit in cancers with high EGFR expression, warranting further investigation in targeted patient populations.

In summary, while several ADCs targeting microtubules have shown promise in preclinical studies and other cancers, their efficacy in glioma treatment remains under investigation.

In the field of neurodegenerative diseases, where reaching vulnerable neurons is a major challenge, antibody-based delivery systems are being explored not only for small molecules but also for transporting therapeutic proteins, liposomes, and enzymes across the BBB [175,176,177]. Although there are still obstacles, particularly achieving consistent BBB penetration and avoiding immune complications, antibody-conjugated drugs represent a powerful, emerging approach for more targeted, efficient, and personalized therapies in both brain cancers and neurodegenerative diseases. The emerging combination strategies aim to stabilize microtubule networks while simultaneously suppressing inflammation, through agents such as tau aggregation inhibitors, HDAC6 inhibitors, or NSAIDs, to preserve neuronal function and slow disease progression [177,178].

Given the multifaceted nature of brain tumours and neurodegenerative diseases, therapeutic resistance and limited efficacy of monotherapies remain significant challenges. As our understanding of microtubule dynamics deepens, combination therapies have emerged as a critical strategy to enhance the therapeutic index of MTAs, particularly in the context of brain tumours and neurodegenerative disorders. By pairing MTAs with agents that improve delivery, target resistance mechanisms, or modulate the tumour microenvironment, researchers are achieving synergistic effects while reducing toxicity. Innovations such as liposomal formulations, antibody–drug conjugates, and dual-targeting compounds exemplify this shift toward more personalized and effective interventions. While challenges persist, especially in translating preclinical success into clinical benefit, combination approaches offer a promising route to overcome the complexity of CNS diseases and improve long-term outcomes.

## 6. Discussion

MTAs hold great promise as a cancer treatment, offering new hope for patients facing various types of cancer, including gliomas. However, their full potential is still held back by some major obstacles, such as systemic toxicity and the development of drug resistance. While drugs like taxanes and vinca alkaloids have shown strong results, they are often limited by side effects (Table 3), especially peripheral neurotoxicity, and struggles with getting the drug to the brain effectively. Recent advancements, such as brain-targeted versions of these drugs and new epothilone derivatives like sagopilone, are helping to reduce side effects while boosting therapeutic potential [114,118]. Current field priorities focus on optimizing MTAs for tubulin isotype/PTM selectivity, improving BBB penetration via nanoparticle or liposomal delivery, and tailoring dosing to avoid neuronal disruption. Practical translation in glioma targeting is evolving beyond mitotic inhibition to disrupting tumour microtube networks, and MTAs are being repurposed for neuroprotection and regeneration in tauopathies and chemotherapy-induced cognitive dysfunction. These developments signal a shift from broad cytotoxicity toward precision microtubule modulation in CNS disease therapy.

Resistance to MTAs continues to be one of the biggest challenges. Drug efflux, driven by transporters like P-glycoprotein, actively pumps the drug out of cells, decreasing its effectiveness [179]. On top of that, changes in tubulin—such as the overexpression of βIII-tubulin—can block the binding of MTAs and reduce their efficacy [180]. While lab studies have shown how certain mutations in tubulin might contribute to resistance, these findings have not yet been confirmed in clinical trials, which shows just how complex and variable resistance can be. Additionally, problems with the cell’s ability to carry out apoptosis, often due to mutations in key proteins like p53 or the upregulation of anti-apoptotic proteins like BCL-XL, allow tumour cells to survive even when microtubules are disrupted [51,181]. Moreover, selectively targeting pathological microtubule dynamics without disrupting essential neuronal functions presents a major therapeutic challenge, particularly in neurodegenerative conditions where neurons are non-dividing but heavily reliant on dynamic microtubule-based transport [182,183].

To get past these barriers, the focus is now on new strategies to make MTAs more targeted and effective. Thanks to advances in structural biology, we are now able to design small-molecule agents that specifically target certain sites on tubulin or MAPs [103], which could minimize unwanted side effects. Another exciting development is the use of drug delivery methods, like attaching MTAs to antibodies or using nanoparticles, which could allow for the more precise delivery of drugs directly to tumours, cutting down on toxicity to healthy tissues [171]. In parallel, a novel therapeutic strategy of indole-based pyridinyl propenones, derived from a known methuosis-inducing compound, highlights a promising avenue for designing dual-mechanism therapeutics that target both methuosis and microtubule dynamics in glioblastoma [184].

Beyond small molecules, RNA-based approaches such as RNA interference (RNAi) and CRISPR-Cas-based RNA editing have emerged as powerful strategies to modulate microtubule-associated genes with high specificity, indirectly or directly. RNAi has been extensively used to silence the expression of tubulin isoforms, MAPs, and regulatory kinases, offering insight into their roles in cancer progression and neuronal degeneration. For instance, in a glioblastoma model (U138MG, U251MG), combining Taxol^®^ with siRNA against a key resistance pathway significantly inhibited cell invasion in vitro [185]. Similarly, targeting stathmin, a microtubule destabilizer, via RNAi impairs glioma cell proliferation and enhances chemotherapy responses [186,187]. More recently, CRISPR-Cas13 and REPAIR-based systems have enabled site-specific editing of RNA transcripts without altering genomic DNA, allowing reversible and programmable interference with microtubule-regulating genes [188,189]. This holds promise in diseases like Alzheimer’s, where post-transcriptional regulation of tau or tubulin isoforms could restore microtubule stability without permanent genome changes. As delivery technologies improve, these RNA-guided methods may complement traditional MTAs by enabling selective, temporally controlled interventions in both cancer and neurodegeneration.

In conclusion, while MTAs face substantial barriers in both efficacy and safety, advances in targeted delivery, molecular design, and gene regulation are steadily transforming their therapeutic potential. As our understanding deepens, MTAs may evolve from broad-spectrum chemotherapeutics into precision tools for treating complex CNS disorders, offering new hope in the fight against brain cancer and neurodegeneration.

**Table 3:** Characteristics of small molecule MTAs for applications in vitro.

Compound	Cell Type/Model	Size (Da)	Working Concentration	Solubility	Half-Life	Known Off-Target Effects	Reference
Paclitaxel	Glioma, neurons	853	1–100 nM	Poor (DMSO required)	~5.8 h (IV)	Neurotoxic	[190,191,192]
Docetaxel	Glioma cells	807	1–50 nM	Low	~11 h	Neurotoxic	[193,194]
Epothilone B	Neurons, tauopathy models	493	0.5–10 nM	Good (aqueous)	~5 h	Mildly neurotoxic	[195,196,197]
Combretastatin A-4	Glioma, endothelial cells	316	1–10 µM	Moderate (DMSO)	~0.4–2 h	Cardiotoxic, hypotension	[198,199,200]
Noscapine	Glioma cells	413	5–100 µM	Good (aqueous)	~2 h	Minimal; low toxicity profile	[201,202]
ABT-751	Pediatric CNS tumours	349	0.1–10 µM	Good (oral bioavailable)	~2–5 h	Neuropathy, GI toxicity	[203,204,205]
TPI-287	Neurons, glioma models	870	10–100 nM	Moderate (formulated)		Peripheral neuropathy	[108,206,207]
Vincristine	Glioblastoma, lymphocytes	825	1–10 nM	Low	~85 h	Neurotoxic, myelosuppression	[208,209,210,211]

## Figures and Tables

**Table 1:** Microtubule-Stabilizing Agents (MSAs) Targeting Tubulin Binding Sites with Relevance to Blood–Brain Barrier Permeability and Brain Pathology Applications.

Agent Name	Tubulin Binding Site	BBB Permeability	Applications in Brain Pathology	Reference(s)
Paclitaxel (Taxol^®^)	Taxane site	Poor (needs vehicle/nanocarriers)	Improved motor function and axonal transport in tau-transgenic mice Used for gliomas and brain metastases	[104,105]
Docetaxel	Taxane site	Poor	Similarly to paclitaxel; limited CNS application unless modified	[106]
Cabazitaxel	Taxane site	Improved over paclitaxel	Studied for CNS delivery; potential in glioblastoma	[107]
TPI-287	Taxane site	Moderate (some BBB penetration)	Phase I trial in glioblastoma (well tolerated) Poor tolerability in Alzheimer’s patients (anaphylactoid reactions)	[108,109,110]
Epothilone B (Patupilone)	Taxane site (non-taxane MSA)	Good	Inhibits glioblastoma cell migration—Limited impact on microtubule dynamics—Preclinical promise in neurodegenerative models	[111,112,113]
Sagopilone	Taxane site (epothilone analogue)	Good	Phase II trial in recurrent glioblastoma (well tolerated, but no antitumour efficacy) Induces apoptosis via microtubule stabilization	[114]
Epothilone D	Taxane site (epothilone analogue)	Good	Alzheimer’s: Enhances axonal microtubule density, reduces dystrophy, improves cognition- Parkinson’s: Reverses axonal damage Stroke/spinal injury: Promotes regrowth	[115,116,117,118,119]
Laulimalide	Laulimalide site (external surface)	Not reported	Strong in vitro anticancer activity No studies in brain tumours—Potential MSA alternative in oncology	[120,121]
Peloruside A	Laulimalide site	Not reported	Restores axonal growth in rat cortex neurons Independent of tau expression—Benefits shown in neuroblastoma cells	[122,123]

**Table 2:** Microtubule-Destabilizing Agents (MDAs) Targeting Tubulin Binding Sites with Relevance to Blood–Brain Barrier Permeability and Brain Pathology Applications.

Agent Name	Tubulin Binding Site	BBB Permeability	Applications in Brain Pathology	Reference
ST-401	Colchicine site	Yes	Brain-penetrant; kills glioblastoma cells in interphase; inhibits tubulin assembly	[144]
PTC596	Colchicine site (distinct binding profile)	Yes	Effective in glioblastoma models; prolongs survival in mice Under trial for DIPG in combination with radiation	[145,146]
RGN3067	Colchicine site	Yes	Effective against glioblastoma in vitro/in vivo; induces G2/M arrest High CNS MPO score	[147]
4AZA2891	Colchicine site (3-nitropyridine class)	Yes (in mice)	Reaches the brain in mice; G2/M arrest; in vitro efficacy shown	[143]
Pironetin	Pironetin site (α-tubulin, Cys316 covalent bind)	Not specified	Induces mitotic arrest and cell death in glioblastoma cell line (T98G) Early-stage anticancer candidate	[148,149,150]
Vincristine	Vinca domain (β-tubulin)	Limited	Widely used for gliomas and medulloblastomas; combination therapy standard	[151,152,153]
Vinblastine	Vinca domain	Limited	Similarly to vincristine; less common in glioma treatment	[154]
Vinorelbine	Vinca domain	Limited	Used in DIPG; combined with nimotuzumab to lower toxicity and improve QoL	[155]
Tumabulin-1 (TM1)	Tumabulin + Colchicine site	Unknown	Dual binding to novel RB3–tubulin interface and colchicine site; dependent on RB3; emerging anticancer strategy	[92,96]
Tumabulin-2 (TM2)	Tumabulin site only	Unknown	Selective binder; enhances RB3-mediated tubulin depolymerization Represents 9th binding site	[92,96]

## Abbreviations

The following abbreviations are used in this manuscript:

ADCs	antibody-drug conjugates
BBB	blood–brain barrier
CNS	central nervous system
DIPG	diffuse intrinsic pontine glioma
GTP	guanosine triphosphate
HDAC	histone deacetylase
MAPs	microtubule-associated proteins
MDAs	microtubule-destabilizing agents
MSAs	microtubule-stabilizing agents
MTAs	microtubule-targeting agents
MTOCs	microtubule organizing centres
PTMs	post-translational modifications
RB3	stathmin-Like Protein B3
RNAi	RNA interference
TM1	Tumabulin-1
TM2	Tumabulin-2
γ-TuRC	γ-tubulin ring complex
